# Transcriptome Analysis of a Female-sterile Mutant (*fsm*) in Chinese Cabbage (*Brassica campestris* ssp. *pekinensis*)

**DOI:** 10.3389/fpls.2017.00546

**Published:** 2017-04-10

**Authors:** Shengnan Huang, Zhiyong Liu, Chengyu Li, Runpeng Yao, Danyang Li, Li Hou, Xiang Li, Wenjie Liu, Hui Feng

**Affiliations:** Department of Horticulture, Shenyang Agricultural UniversityShenyang, China

**Keywords:** Chinese cabbage, female-sterile mutant, pistil development, RNA-Seq technology, DEGs

## Abstract

Female-sterile mutants are ideal materials for studying pistil development in plants. Here, we identified a female-sterile mutant *fsm* in Chinese cabbage. This mutant, which exhibited stable inheritance, was derived from Chinese cabbage DH line ‘FT’ using a combination of isolated microspore culture and ethyl methanesulfonate mutagenesis. Compared with the wild-type line ‘FT,’ the *fsm* plants exhibited pistil abortion, and floral organs were also relatively smaller. Genetic analysis indicated that the phenotype of *fsm* is controlled by a single recessive nuclear gene. Morphological observations revealed that the presence of abnormal ovules in *fsm* likely influenced normal fertilization process, ultimately leading to female sterility. Comparative transcriptome analysis on the flower buds of ‘FT’ and *fsm* using RNA-Seq revealed a total of 1,872 differentially expressed genes (DEGs). Of these, a number of genes involved in pistil development were identified, such as *PRETTY FEW SEEDS 2* (*PFS2*), *temperature-induced lipocalin* (*TIL*), *AGAMOUS-LIKE* (*AGL*), and *HECATE* (*HEC*). Furthermore, GO and KEGG pathway enrichment analyses of the DEGs suggested that a variety of biological processes and metabolic pathways are significantly enriched during pistil development. In addition, the expression patterns of 16 DEGs, including four pistil development-related genes and 12 floral organ development-related genes, were analyzed using qRT-PCR. A total of 31,272 single nucleotide polymorphisms were specifically detected in *fsm*. These results contribute to shed light on the regulatory mechanisms underlying pistil development in Chinese cabbage.

## Introduction

Floral organ development is the most obvious characteristic of the reproductive stage of flowering plants. The flowers of typical dicotyledonous plants are composed of four wheel-like structures (whorls). The first whorl (from outside to inside) contains the sepals, the second contains the petals, the third contains the stamens, and the innermost whorl contains the carpels, which are the female organs. In recent years, a variety of floral organ mutants have been characterized, leading to the isolation of a series of floral development- and morphogenesis-related genes using various techniques, and the expression patterns and functions of these genes have also been analyzed (Hu et al., 2003; Szécsi et al., 2006; Koyama et al., 2007; Nag et al., 2009; Sarvepalli and Nat, 2011; Lu et al., 2012; Huang et al., 2015). Studies on the commonality and characteristics of floral development in different species can help elucidate the origins and evolution of flowers. Such information would lay the foundation for altering the process of floral development, which might be used to control the flowering process and fertility in plants.

Female sterility has been identified in crops such as wheat (*Triticum aestivum*) (Dou et al., 2001), rice (*Oryza sativa*) (Tang et al., 2002), soybean (*Glycine max*) (Pereira et al., 1997), pearl millet (*Pennisetum glaucum*) (Arthur et al., 1993), ramie (*Boehmeria nivea*) (Zhou, 1996), and rapeseed (*Brassica napus*) (Chen et al., 2003). Studies have been performed on the biological characteristics, development, genetic methods, and potential applications of female sterility (Brown and Bingham, 1984; Luo et al., 1996; Daskalov and Mihailov, 1998; Xiao et al., 1998; Zhong et al., 1998; Li and Zheng, 2002; Liu et al., 2003; Sun et al., 2009). Female-sterile mutants have been used to identify regulatory genes that influence ovule and female gametophyte development. These regulatory genes are involved in nucellar and integument cell development (Schiefthaler et al., 1999; Balasubramanian and Schneitz, 2000, 2002), the regulation of megasporogenesis (Schneitz et al., 1997; Singh et al., 2011; Chevalier et al., 2013), metabolism, division and differentiation, and the developmental regulation of embryo sac cells (Pagnussat et al., 2005; Portereiko et al., 2006; Johnston et al., 2007; Jones-Rhoades et al., 2007; Colombo et al., 2008; Moll et al., 2008; Punwani et al., 2008).

The female-sterile mutants (with abnormal ovule and embryo sac development) have recently been produced through mutagenesis, including chemical mutagenesis, transposon mutagenesis, and T-DNA insertion mutagenesis (Bao et al., 2005; Venkatesan and Monica, 2010). Mutation of genes controlling female organ development, such as carpel development gene *DROPPING LEAF* (*DL*) and ovule development genes *FLORAL BINDING PROTEIN 7* (*FBP7*) and *SHATTERPROOF 1* (*SHP1*) (Cheng et al., 2000; Ferrándiz et al., 2000; Yamaguchi et al., 2004), can lead to female sterility. In addition, such mutations can lead to abnormal ovule or endosperm development, thereby affecting the function of female gametophytes as well as megasporogenesis (Huang and Sheridan, 1996; Siddiqi et al., 2000; Shi et al., 2005). Luo et al. (2001) found that the abnormal pistil development trait in rice mutant *dl(t)* is controlled by a single recessive gene, whose function may be similar to that of *SUPERMAN* (*SUP*), which regulates floral organ development in *Arabidopsis thaliana* (Sakai et al., 1995). The *SUP* inhibits the expression of floral organ development genes in the pistil, functioning as a cadastral gene of stamen and pistil genes (Jacobsen and Meyerowitz, 1997; Nibau et al., 2011). These mutants would be useful for studying female sterility-related proteins, cloning the related genes, and further analyzing their structures and functions, shedding light on the molecular mechanisms underlying sex differentiation and development in floral organs.

The male-sterile line has played an important role in the plant hybrid breeding. The research showed that the female-sterile line can also be used as the pollinator, the pollination distance between the both parents can be reduced, thus improving the hybrid seed yield. Therefore, further studies of female-sterile mutants not only help uncover additional information about floral organ development, but also may provide important basic materials for hybrid breeding (Maruyama et al., 1991; Dou et al., 2001).

Transcriptome analysis, i.e., investigating transcription and the regulation of all genes in an organism at the genome-wide level, is an important component of functional genomics research (Qi et al., 2011; Li et al., 2013). Transcriptome analysis can uncover the global expression patterns of genes and provide information about gene-protein interactions in plants (Filichkin et al., 2010; Montgomery et al., 2010; Faulconnier et al., 2011; Li et al., 2012; Ren et al., 2012; Zhang et al., 2012; Huang et al., 2015). The recent transcriptional profiling studies on the female gametophyte development have demonstrated that the female gametophyte formation is a complicated process, and numerous genes are involved in the regulation of female gametophyte formation in several plant species (Pagnussat et al., 2005; Anderson et al., 2013; Chettoor et al., 2014). Therefore, the female-sterile mutants are ideal for revealing the molecular mechanism of pistil development, and further analyses of the gene expression changes during pistil development are very necessary.

Chinese cabbage (*Brassica campestris* ssp. *pekinensis* [Lour] Olsson), an economically and nutritionally important vegetable crop, is widely cultivated in Northeast Asia. With the completion of genome sequencing of Chinese cabbage (Wang et al., 2011), transcriptome analysis of this crop has become an important field of study. Considering that the female-sterile plants are a powerful tool to study genes involved in the pistil development and investigate gene functions (Arthur et al., 1993; Rosellini et al., 1998, 2003), and thus the female-sterile mutants may be useful for the study of the reproductive system of Chinese cabbage.

In this study, we identified a female-sterile mutant (*fsm*) in Chinese cabbage. To help elucidate the molecular mechanism underlying pistil development, we conducted comparative transcriptome analysis using the Illumina sequencing platform HiSeq^TM^ 2000 to characterize the gene expression profiles in flower buds of *fsm* and the corresponding wild-type line ‘FT’ on a global level. The main objective of this study was to identify differentially expressed genes (DEGs) and potential candidate genes related to pistil development. Our results provide a comprehensive view of the transcriptome of Chinese cabbage, which contributes to increase our understanding of the regulatory mechanisms underlying pistil development in this crop.

## Materials and Methods

### Plant Materials and Mutagenic Treatment

The *fsm* mutant was derived from a Chinese cabbage doubled-haploid (DH) line ‘FT’ via a combination of isolated microspore culture and ethyl methanesulfonate (EMS) mutagenesis. Based on our parallel study (Huang et al., 2016a), the isolated microspores were treated with 0.08% EMS solution for 10 min. The microspore regenerated plants were transplanted to pots and cultivated in the greenhouse for further growth and development.

It is thought that the genetic background between ‘FT’ and *fsm* is highly consistent, with the difference mainly occurring at the mutation sites.

### Morphological Observation

In December 2014, the seeds of ‘FT’ and *fsm* were sown in a greenhouse at Shenyang Agricultural University, China. In March 2015, morphological analyses of ‘FT’ and *fsm* plants were carried out at the full-bloom stage.

#### Floral Organs Observation

Four-wheeled floral organs were directly observed and photographed under a dissecting microscope (Nikon SMZ800, Japan).

#### Identification of Female Sterility

Three ‘FT’ and *fsm* plants were respectively selected, and bagged for 3 days before pollination. Twenty flower buds were randomly selected from each plant, and the artificial self-pollination of ‘FT’ and *fsm* was carried out. Additionally, 20 flower buds were randomly selected from each plant again, and a reciprocal cross between ‘FT’ and *fsm* was performed. After the seeds were mature, the seed setting rates were recorded.

#### Pollen Viability Detection

The anthers were removed from the stamens and the pollen from each anther was extruded onto a slide. The pollen was immersed in 0.1% TTC (2,3,5-triphenyltetrazolium chloride) dye solution, covered with a cover slip, and dyed for 15–20 min at 35–37°C in an incubator. Pollen viability was observed under an optical microscope (Nikon ECLIPSE 80i, Japan).

#### Ovary and Ovule Development Observation

Ten flower buds were randomly selected from ‘FT’ and *fsm* plants, respectively. Flowers on the first flowering day of ‘FT’ and *fsm* were respectively marked and artificially pollinated. The length and width of the ovaries were measured every other day; ovaries were measured six times, and each measurement was performed in three independent experiments. The ovules in the ovary of ‘FT’ and *fsm* on the first flowering day and the 5th day after pollination were respectively observed and compared under a dissecting microscope (Nikon SMZ800, Japan); Accordingly, the pistils on the first flowering day and the 5th day after pollination were fixed using FAA (Formalin-Aceto-Alcohol) fixative and then dyed using safranine and fast green. The detailed procedures of paraffin section were performed according to the traditional method of Li (1996), and the ovule development was observed under an optical microscope (Nikon ECLIPSE 80i, Japan).

### Genetic Analysis

To investigate the inheritance of *fsm*, ‘FT’ (P_1_) and *fsm* (P_2_) were used as the parents to develop the F_1_, BC_1_, and F_2_ populations. Phenotypic data were obtained for each plant of the P_1_, P_2_, F_1_, BC_1_, and F_2_ populations, and the segregation ratios of the BC_1_ and F_2_ populations were analyzed by a Chi-square (χ^2^) test.

### RNA Extraction

In March 2015, developing flower buds were collected from ‘FT’ and *fsm* at the full-bloom stage and used as transcriptome sequencing materials. All samples were immediately frozen in liquid nitrogen and stored at -80°C.

At the full-bloom stage, five ‘FT’ and *fsm* plants were respectively selected, and three inflorescences were randomly selected from each plant. All the flower buds from these inflorescences collected from five ‘FT’ and *fsm* plants were respectively mixed, and the mixed samples were used as a single biological replicate; three independent biological replicates were performed for ‘FT’ and *fsm*.

Total RNA from six samples of ‘FT’ and *fsm* (with three independent biological replicates) was respectively extracted using TRIzol reagent (Invitrogen, USA) following the manufacturer’s instructions, and the DNase treatment was applied during RNA extraction to reduce DNA contamination. The quality and integrity of all RNA samples were assessed with a 2100 Bioanalyzer (Agilent Technologies, USA) and by electrophoresis on 1.0% agarose gels.

### cDNA Library Construction and Illumina Sequencing

To construct six cDNA libraries, equal amounts of total RNA from the three independent biological replicates of ‘FT’ and *fsm* were pooled for RNA-Seq library construction, which were designated F1, F2, F4, M1, M2, and M3, respectively. Oligo (dT)-coated magnetic beads were used to isolate mRNA, which was broken into small fragments by the addition of fragmentation buffer. First-strand cDNA was synthesized with these short fragments serving as templates, and second-strand cDNA was synthesized using the reaction system. The short fragments were purified and subjected to end repair and the addition of sequencing adapters. Following agarose gel electrophoresis, suitable fragments were selected as templates for PCR amplification. Quantification and quality analysis of the constructed libraries were conducted using an Agilent 2100 Bioanalyzer and an ABI StepOnePlus Real-Time PCR System (Li et al., 2011; Huang et al., 2016b). The cDNA libraries were then sequenced on Illumina sequencing platform HiSeq^TM^ 2000 with a paired-end sequencing strategy at Beijing Genomics of Institute (BGI), Shenzhen, China.

### Mapping of Reads to the Reference Genome

Prior to bioinformatic analysis, the raw image data were transformed into sequence data by base calling. Clean reads were obtained by removing reads containing adaptors, reads with more than 10% unknown nucleotides, and low-quality reads with more than 50% bases with a quality value < 20. Clean reads were mapped to the reference genome (Version 1.5)^1^ with SOAPaligner/SOAP2 (Li et al., 2009b), allowing no more than five base mismatches in the alignment.

### Identification of SNPs

The genotype of three biological replicates of ‘FT’ and *fsm* were identical. Therefore, RNA-Seq data from the three biological replicates of ‘FT’ and *fsm* were combined for single nucleotide polymorphism (SNP) identification, respectively. In this study, SNPs were identified using SOAPsnp software (Li et al., 2009a). The SOAPsnp program is a resequencing utility that can detect the consensus sequence for the transcriptome of a sequencing individual based on the alignment of the sequencing reads on the known reference sequence. The SNPs can then be identified on the consensus sequence through the comparison with the reference sequence.

### Assessment of Differential Gene Expression

The expression levels of genes determined by RNA-Seq were normalized by the RPKM (reads per kb per million mapped reads) method, thereby limiting the effects of different gene lengths and sequencing levels on the calculation of gene expression level (Mortazavi et al., 2008). DESeq was applied to identify DEGs based on the RPKM-derived baseMean for each gene between samples (Anders and Huber, 2010). The false discovery rate (FDR) was used as the threshold of *P-*value in multiple tests (Benjamini and Hochberg, 1995). The combination of FDR ≤ 0.001 and the absolute value of log_2_Ratio ≥ 1 were used as the threshold for judging the significance of differences in gene expression (Benjamini and Yekutieli, 2001). More stringent criteria, including smaller FDR and larger fold-change values, can be used to identify DEGs. In the current study, genes with FDR ≤ 0.001 and the absolute value of log_2_ Ratio ≥ 4 were defined as DEGs. In addition, the specifically expressed genes (SEGs) were detected, i.e., genes that were not expressed in one library but had baseMean values ≥ 11 in the other library (Tao et al., 2012).

### Functional Enrichment Analysis of DEGs

To characterize the biological functions and metabolic pathways of the DEGs, the DEGs were subjected to Gene Ontology (GO)^2^ functional analysis (Ashburner et al., 2000) and Kyoto Encyclopedia of Genes and Genomes (KEGG)^3^ pathway enrichment analysis (Kanehisa et al., 2008). Compared to the genome background, the significantly enriched GO terms and KEGG pathways for the DEGs were determined using hypergeometric tests, with the Bonferroni-corrected *P*-value ≤ 0.05 and Q value ≤ 0.05 as the thresholds, respectively (Abdi, 2007).

### Quantitative Real-time PCR (qRT-PCR) Analysis

Total RNA was extracted from the same plant samples of ‘FT’ and *fsm* as those used for RNA-Seq using TRIzol reagent (Invitrogen, USA), and cDNA was synthesized using a FastQuant First-strand cDNA Synthesis kit (Tiangen, Beijing, China) according to the manufacturer’s instructions. The *Actin* and *18S rRNA* were used as internal reference controls (Huang et al., 2015; Chen et al., 2016) and gene-specific primers were designed using Primer Premier 5.0 software. The qRT-PCR analysis was carried out using SYBR Green as a fluorescent detection dye (Tiangen, Beijing, China) and performed on a Bio-Rad IQ5 real time PCR detection system (Bio-Rad, USA). Each reaction contained 9 μl 2.5× Real MasterMix/20× SYBR solution, 2 μL (2 μmol L^-1^) of each forward and reverse primers, 2 μl of diluted cDNA (50 ng), and 5 μl ddH_2_O to a final volume of 20 μL. The qRT-PCR program was performed in 96-well plates under the following cycling conditions: initial activation at 95°C for 3 min, followed by 40 cycles of 95°C for 30 s, 58°C for 30 s, and 68°C for 15 s. This procedure was followed by melting curve analysis from 55 to 95°C to check the specificity of PCR amplification. The 2^-ΔΔCt^ method was employed to calculate the relative expression levels of the target genes (Livak and Schmittgen, 2001). All reactions were performed with three biological and technical replicates, respectively. Differences in gene expression were analyzed using Bio-Rad IQ5 Manager software.

## Results

### Identification and Genetic Analysis of *fsm*

A large number of microspore regenerated plants (M_0_ generation) were obtained using the isolated microspore culture combined with EMS mutagenesis treatment, and the double haploid plants were screened for investigating botanical characteristics. The variant plants were observed and all double haploid plants were selfed in the M_0_ generation. In the M_1_ generation, the variant traits and genetic stability of mutants were further identified.

The *fsm* mutant exhibited the same visible phenotype as wild-type line ‘FT’ in the M_0_ generation. After selfing, segregation of characters appeared in the M_1_ generation (segregation ratio of 196: 54): of the 250 plants, 54 plants showed pistil abortion; other plants with the same phenotype as ‘FT’ were further selfed, revealing that character segregation continued in the offspring. These results suggest that the mutation may have occurred during the spontaneous diploid period rather than during the haploid period during the process of microspore culture and that the mutant gene *fsm* is recessive.

Therefore, to further investigate the inheritance of *fsm*, ‘FT’ (P_1_) and *fsm* (P_2_) were used as the parents. As shown in **Table 1**, the ‘FT’: *fsm* ratio among the BC_1_ progenies produced from the F_1_ × *fsm* backcross was approximately 1: 1 (χ^2^ = 2.07 < χ^2^_0.05,1_ = 3.84). Of the 226 F_2_ plants, 178 and 48 individuals showed the ‘FT’ and *fsm* phenotypes, respectively, which represents a segregation ratio of 3.71: 1. The segregation ratios in the F_2_ population conformed to the expected ratio of 3: 1 (χ^2^ = 1.81 < χ^2^_0.05,1_ = 3.84). These results indicate that the phenotype of *fsm*, which exhibiting stable inheritance, is controlled by a single recessive nuclear gene.

**Table 1:** Genetic analysis of *fsm* and crosses between *fsm* and wild-type line ‘FT.’

Generation	‘FT’	*Fsm*	Total	Segregation ratio	Expected ratio	χ^2^
P_1_ (‘FT’)	105	0	105			
P_2_ (*fsm*)	0	54	54			
F_1_ (P_1_ × P_2_)	170	0	170			
F′_1_ (P_2_ × P_1_)	0	0	0			
BC_1_ (F_1_ × ‘FT’)	206	0	206			
BC_1_ (F_1_ × *fsm*)	97	78	175	1.24: 1	1:1	2.07
F_2_	178	48	226	3.71: 1	3:1	1.81

### Morphological Characteristics of Floral Organs in ‘FT’ and *fsm*

**FIGURE 1:**
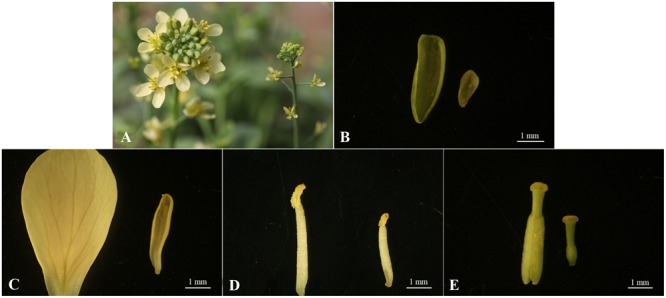
**Morphological characteristics of floral organs from *fsm* and wild-type line ‘FT.’ (A)** Flowers of ‘FT’ (left) and *fsm* (right); **(B)** sepals of ‘FT’ (left) and *fsm* (right); **(C)** petals of ‘FT’ (left) and *fsm* (right); **(D)** stamens of ‘FT’ (left) and *fsm* (right); **(E)** pistils of ‘FT’ (left) and *fsm* (right). Scale bar: 1 mm.

Compared to the wild-type line ‘FT,’ the *fsm* plants exhibited pistil abortion, and the four-wheeled floral organs were also relatively smaller (**Figure 1**). As shown in **Figure 1E**, the pistil parts in *fsm* were significantly thinner and shorter, especially the ovaries.

### Female Sterility Analysis of *fsm*

As shown in **Table 2**, compared to the wild-type line ‘FT,’ the *fsm* plants exhibited pistil abortion. Whether the *fsm* mutant was self-pollinated or used as the female parent to accept foreign pollen (wild-type line ‘FT’), the seed setting rates of *fsm* were both zero. The results showed that the female sterility of *fsm* was stable.

**Table 2:** The seed setting rates of self-pollination and reciprocal crosses between *fsm* and wild-type line ‘FT.’

Generation	No. of pollinated flower buds	No. of harvested seeds	No. of seeds per bud
*fsm*⊗	60	0	0
‘FT’ ⊗	60	976	16.27
*fsm* × ‘FT’	60	0	0
‘FT’ × *fsm*	60	628	10.47

### Pollen Viability Observation of *fsm*

**FIGURE 2:**
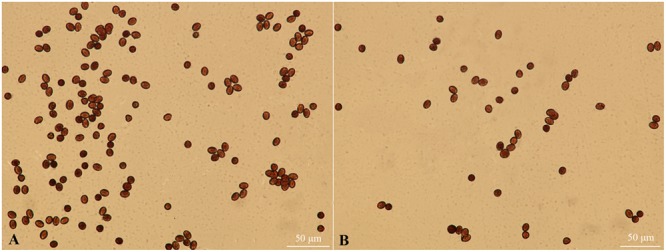
**Pollen viability observation of *fsm* and wild-type line ‘FT.’ (A)** Mature pollen microspores of ‘FT’; **(B)** mature pollen microspores of *fsm*. Scale bar: 50 μm.

The pistils of *fsm* were completely sterile; however, there were small amounts of pollen in the stamens. As shown in **Figure 2**, the pollen of *fsm* was viable. In accordance with the results of **Table 2**, therefore, the fertility of *fsm* stamens was normal.

### Morphological Comparison of Ovary and Ovule Development in ‘FT’ and *fsm*

**FIGURE 3:**
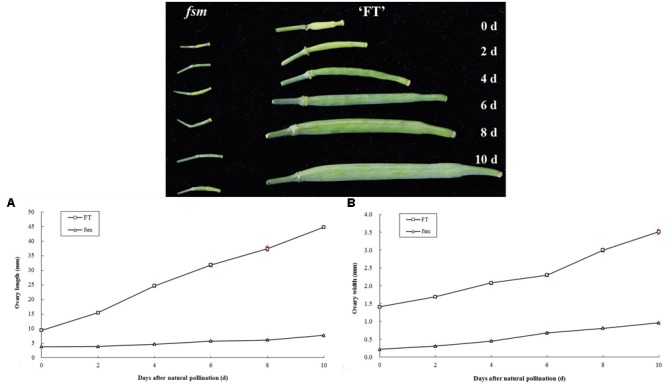
**Ovary development in *fsm* and wild-type line ‘FT’ on different days after artificial pollination. (A)** Dynamic changes of ovary length; **(B)** dynamic changes of ovary width. Each value is the mean of three independent experiments. The *error bars* represent standard error (SE) of the means.

In plants, the development of female reproductive organs meant the ovary development, mainly including the development of ovule and formation of embryo sac. To further investigate the female sterility phenotype of *fsm*, the ovary and ovule development in ‘FT’ vs. *fsm* were observed and compared. As shown in **Figure 3**, under artificial pollination conditions, ovary development in *fsm* stopped at the end of flowering. The ovaries gradually became atrophied and yellow, ultimately leading to abscission. By contrast, the ovaries of ‘FT’ were elongated and widened at the end of flowering, and they developed rapidly.

**FIGURE 4:**
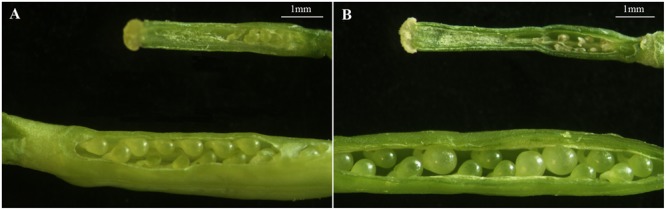
**Structural characteristics of ovules in *fsm* and wild-type line ‘FT.’ (A)** Ovules on the first flowering day in *fsm* (above) and ‘FT’ (below); **(B)** ovules of the 5th day after pollination in *fsm* (above) and ‘FT’ (below). Scale bar: 1 mm.

**FIGURE 5:**
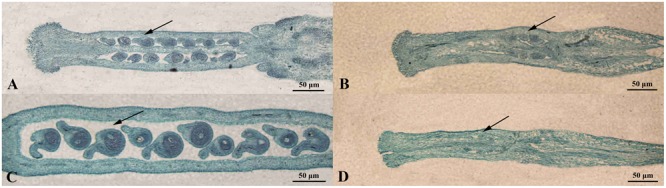
**Morphological comparison of ovule development in *fsm* and wild-type line ‘FT.’ (A)** Ovules on the first flowering day in ‘FT’; **(B)** ovules on the first flowering day in *fsm*; **(C)** ovules of the 5th day after pollination in ‘FT’; **(D)** ovules of the 5th day after pollination in *fsm*. Scale bar: 50 μm.

Also, the structural characteristics of ovule development were observed and compared in ‘FT’ and *fsm*. As shown in **Figure 4**, compared with ‘FT,’ the ovules of *fsm* were abnormal, fewer, and smaller. After artificial pollination, the ovules of ‘FT’ were expanded and eventually developed into seeds, whereas the ovules of *fsm* were shriveled and did not develop into seeds. The observation results were in accordance with the results of paraffin section (**Figure 5**).

### Illumina Sequencing and Mapping Reads to the Reference Genome

To help increase our understanding of the regulatory mechanisms underlying pistil development at the molecular level, we performed comparative floral transcriptome analysis of ‘FT’ and *fsm* using RNA-Seq technology.

Based on Illumina sequencing, a total of 130,216,688 and 134,955,118 clean reads were generated from the three biological replicates of ‘FT’ and *fsm*, respectively. Of the total clean reads, the number of reads that could be mapped to the reference genome ranged from 23.3 to 28.3 million, and the percentage of cleans reads ranged from 53.95 to 61.52% in the six libraries. As shown in **Table 3**, the overwhelming majority of these mapped reads were matched to unique genomic locations. The uniquely matched reads were used for gene expression analysis between ‘FT’ and *fsm*. These transcriptomes provide valuable resources for further analysis.

**Table 3:** Reads statistics based on RNA-Seq data of six libraries from *fsm* and wild-type line ‘FT.’

Summary	F1	F2	F4	M1	M2	M3
Total clean reads	43,518,072	43,433,878	43,264,738	46,054,444	44,645,322	44,255,352
Total base pairs	5,439,759,000	5,429,234,750	5,408,092,250	5,756,805,500	5,580,665,250	5,531,919,000
Total mapped reads	23,522,184 (54.05%)	23,450,417 (53.99%)	23,339,692 (53.95%)	28,329,921 (61.51%)	27,464,883 (61.52%)	27,164,647 (61.38%)
Perfect match reads	13,181,506 (30.29%)	13,175,823 (30.34%)	13,074,384 (30.22%)	15,992,485 (34.73%)	15,568,851 (34.87%)	15,373,270 (34.74%)
≤5 bp mismatch reads	10,340,678 (23.76%)	10,274,594 (23.66%)	10,265,308 (23.73%)	12,337,436 (26.79%)	11,896,032 (26.65%)	11,791,377 (26.64%)
Unique match reads	22,821,592 (52.44%)	22,606,654 (52.05%)	22,581,156 (52.19%)	27,684,983 (60.11%)	26,845,981 (60.13%)	26,548,687 (59.99%)
Multi-position match reads	700,592 (1.61%)	843,763 (1.94%)	758,536 (1.75%)	644,938 (1.40%)	618,902 (1.39%)	615,960 (1.39%)
Total unmapped reads	19,995,888 (45.95%)	19,983,461 (46.01%)	19,925,046 (46.05%)	17,724,523 (38.49%)	17,180,439 (38.48%)	17090705 (38.62%)

*Total mapped reads are the sum of perfectly match reads and reads with **≤**5 bp mismatch, or the sum of uniquely matched reads and multi-position matched reads. Numbers in parentheses indicate the percentages of total clean reads in each library.*

**FIGURE 6:**
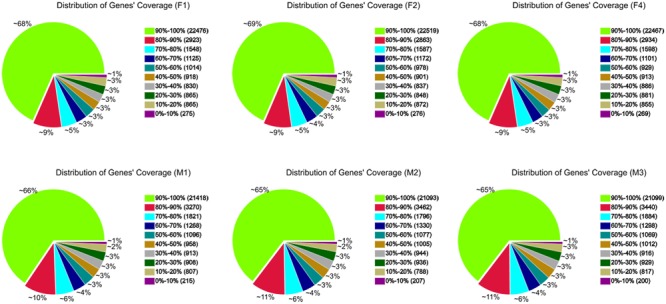
**Distribution of gene coverage in *fsm* and wild-type line ‘FT.**’

A total of 36,120 genes were detected in ‘FT’ and *fsm*. Among these, 32,843 (F1), 32,853 (F2), 32,837 (F4), 32,675 (M1), 32,640 (M2), and 32,665 (M3) expressed genes were identified from the six libraries, respectively (**Supplementary Table S1**). To further evaluate the RNA-Seq data, we analyzed the distribution of gene coverage in each library, representing the percentage of a gene covered by reads. As shown in **Figure 6**, genes with coverage > 90% were the most abundant category, accounting for 65–69% of the total number of genes. The second most abundant category was gene coverage of 80–90%, while the percentages of gene coverage for the remaining eight categories were similar.

### Identification of SNPs

A total of 167,837 and 155,448 SNPs were identified in ‘FT’ and *fsm*, respectively (**Supplementary Tables S2, S3**). As shown in **Table 4**, the most common base substitutions were A/G and C/T, and the least common was C/G. In addition, the SNPs between ‘FT’ and *fsm* were further analyzed and compared, among these SNPs, 43,661 and 31,272 SNPs were specifically detected in ‘FT’ and *fsm*, respectively (**Supplementary Tables S4, S5**).

**Table 4:** Summary of single nucleotide polymorphism (SNP) types identified in *fsm* and wild-type line ‘FT.’

SNP type	‘FT’	*fsm*
Transition	93,291 (55.58%)	89,372 (57.49%)
A/G	46,987 (28.00%)	43,980 (28.29%)
C/T	46,304 (27.59%)	45,392 (29.20%)
Transversion	74,546 (44.42%)	66,076 (42.51%)
A/C	19,933 (11.88%)	18,010 (11.59%)
A/T	19,551 (11.65%)	16,648 (10.71%)
C/G	15,990 (9.53%)	14,282 (9.19%)
G/T	19,072 (11.36%)	17,136 (11.02%)
Total	167,837	155,448

*Numbers in parentheses indicate the percentages of total number of SNPs in ‘FT’ and *fsm*, respectively.*

### Global Analysis of Differential Gene Expression

Comparative analysis of the gene expression profiles between ‘FT’ and *fsm* were conducted to identify DEGs. A total of 1,872 DEGs were detected, including 1,021 up-regulated and 851 down-regulated genes in the *fsm* vs. ‘FT’ comparison, respectively. Therefore, the number of up-regulated DEGs in *fsm* is higher than the number of down-regulated DEGs (**Supplementary Table S6**).

We also detected a number of SEGs in this study. A total of 178 SEGs were identified between ‘FT’ and *fsm*, including 49 SEGs in ‘FT’ and 129 in *fsm* (**Supplementary Table S7**).

### DEGs Related to Pistil Development

The morphological characterization suggested that the presence of the mutant gene *fsm* likely influenced pistil development (especially the presence of abnormal ovules), and thus the fertilization process cannot be accomplished, ultimately leading to female sterility.

To identify potential genes related to pistil development, we compared the gene expression profiles of ‘FT’ and *fsm*. Among the DEGs, a number of pistil development-related genes were identified, including genes for *PRETTY FEW SEEDS 2* (*PFS2*; *Bra026791*), *temperature-induced lipocalin* (*TIL*; *Bra020391*), *AGAMOUS-LIKE* (*AGL*; *Bra029154*), and *HECATE* (*HEC*; *Bra012128*). In the *fsm* vs. ‘FT’ comparison, these genes were up-regulated, with relatively high expression levels (**Supplementary Table S6**), providing clues about the molecular mechanisms underlying female sterility.

The *fsm* mutant not only exhibited pistil abortion, but the floral organs were also relatively smaller compared to the wild-type line ‘FT’ (**Figure 1**). Consequently, numerous DEGs involved in floral organ development were also identified in this study, such as genes encoding F-box family protein (*Bra001764, Bra004091, Bra027182*, and so on), JASMONATE-ZIM-DOMAIN PROTEIN (JAZ; *Bra022981, Bra025713*, and *Bra031065*), *VANGUARD 1* (*VGD 1*; *Bra000438* and *Bra040474*), *FLOWERING LOCUS T* (*FT*; *Bra022475*), polygalacturonase (PG; *Bra001268, Bra001269, Bra025631*, and so on), *MYB* family transcription factors (*Bra012579, Bra013526*, and *Bra028717*), Arabinogalactan proteins (AGPs; *Bra003296, Bra014611, Bra016902*, and so on), *EARLY FLOWERING 4-LIKE 1* (*EFL1*; *Bra000468*), pectinesterase family protein (*Bra009264, Bra009921, Bra034960*, and so on), *BROTHER OF FT AND TFL1* (*TERMINAL FLOWER 1*) protein (*BFT*; *Bra010052*), especially for *Auxin-Regulated Gene Involved In Organ Size* (*ARGOS*; *Bra007491*), which can regulate the floral organ size in *Arabidopsis thaliana* (Hu et al., 2003). Most of these genes were highly expressed in the *fsm* vs. ‘FT’ comparison (**Supplementary Table S6**).

Overall, these genes may play important roles in floral organ development in Chinese cabbage. Further investigating possible pistil development-related genes would help elucidate the gene expression patterns and regulatory mechanisms involved in the female sterility phenotype of *fsm*.

### Functional Enrichment Analysis of DEGs Using GO Classification and KEGG Pathway Analysis

**FIGURE 7:**
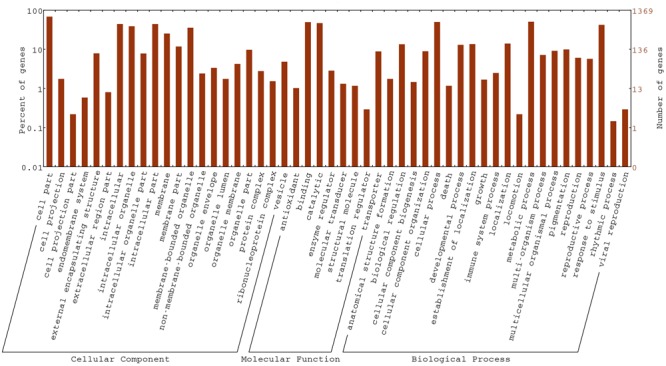
**Gene ontology (GO) functional classification of DEGs in the *fsm* vs. ‘FT’ comparison**.

To gain insight into the biological functions of the DEGs, all DEGs in the *fsm* vs. ‘FT’ comparison were mapped to GO terms using GO functional category analysis. For the three main GO categories, DEGs assigned to “biological process” (1,084, 57.9%) accounted for the majority of genes, followed by “molecular function” (1,077, 57.5%) and “cellular component” (960, 51.3%). Among these, the terms “cellular process” (GO: 0009987) and “metabolic process” (GO: 0008152), with 648 genes (59.8%) and 669 genes (61.7%), respectively, were dominant in the biological process category. In the molecular function category, the terms “binding” (GO: 0005488; 677, 62.9%) and “catalytic activity” (GO: 0003824; 638, 59.2%) were the most highly represented. In the cellular component category, “cell” (GO: 0005623; 780, 81.2%), “cell part” (GO: 0044464; 780, 81.2%), and “intracellular” (GO: 0005622; 606, 63.1%) were the most abundant groups (**Figure 7**).

We also performed GO term enrichment analysis (corrected *P*-value ≤ 0.05). The significantly enriched GO terms were shown in **Supplementary Table S8**. In addition, GO analysis revealed a number of GO terms related to floral organ development, including gynoecium development (*Bra012128* and *Bra000979*), pollen tube growth (*Bra037213* and *Bra034771*), floral organ morphogenesis (*Bra012128, Bra000979* and *Bra026791*), pollen tube development (*Bra012579, Bra037213*, and *Bra034771*), floral organ formation (*Bra012128* and *Bra000979*), floral organ development (*Bra012128, Bra001005, Bra000979, Bra026791, Bra033931*, and *Bra012639*), floral whorl development (*Bra012128* and *Bra000979*), pollen development (*Bra037213, Bra003255*, and *Bra020195*), and flower development (*Bra012639, Bra012128, Bra001005, Bra000979, Bra026791*, and *Bra033931*, and so on).

To identify genes involved in metabolic or signal transduction pathways, a total of 949 DEGs were mapped to 226 KEGG pathways. Metabolic pathways (ko01100; 218, 22.97%) was the largest category, which was significantly larger than other pathways, followed by biosynthesis of secondary metabolites (ko01110; 131, 13.80%), plant-pathogen interaction (ko04626; 126, 13.28%), plant hormone signal transduction (ko04075; 103, 10.85%), and protein processing in endoplasmic reticulum (ko04141; 51, 5.37%). In addition, the KEGG enrichment analysis of DEGs was performed in this study. As shown in **Table 5**, a total of eight KEGG pathways were significantly enriched. These results indicated that a variety of genetic and active metabolic pathways were involved in pistil development, which laid the foundation for further investigating specific processes, functions, and pathways underlying female sterility in Chinese cabbage.

**Table 5:** Significantly enriched KEGG pathways of DEGs in *fsm* vs. ‘FT.’

Pathway	DEGs (%)	Total DEGs	Q-value	Pathway ID
Metabolism of xenobiotics by cytochrome P450	19 (2%)	949	1.06E-05	Ko00980
Plant-pathogen interaction	126 (13.28%)	949	4.72E-05	Ko04626
Drug metabolism – cytochrome P450	19 (2%)	949	5.16E-05	Ko00982
Protein processing in endoplasmic reticulum	51 (5.37%)	949	2.36E-03	Ko04141
Carotenoid biosynthesis	24 (2.53%)	949	2.36E-03	Ko00906
Glutathione metabolism	20 (2.11%)	949	2.74E-03	Ko00480
Antigen processing and presentation	18 (1.9%)	949	2.45E-02	Ko04612
Plant hormone signal transduction	103 (10.85%)	949	2.45E-02	Ko04075

*DEGs: number of DEGs with specific pathway annotations. Total DEGs: total number of DEGs with pathway annotations. Percentage (%) = 100% × (number of DEGs)/total number of DEGs.*

### Analysis of the Gene Expression Patterns by qRT-PCR

**FIGURE 8:**
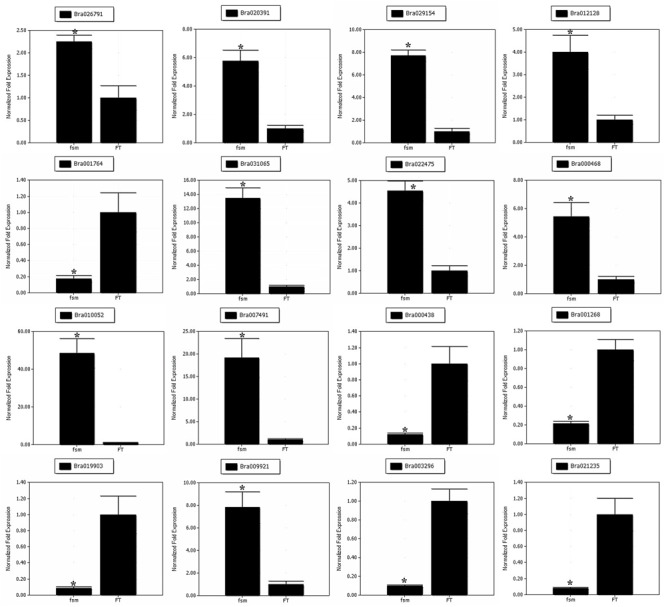
**Quantitative real-time polymerase chain reaction (qRT-PCR) analysis of gene expression patterns**. The relative expression levels of 16 DEGs identified by RNA-Seq analysis are shown. The gene expression analysis was performed based on three biological and technical replicates, respectively. ^∗^Significantly different at a level of 0.05 by *t*-test, and the statistical analysis was conducted using SPSS16.0 (Chicago, IL, USA).

To help confirm the differential expression patterns of the DEGs detected by RNA-Seq, we performed qRT-PCR analysis of various DEGs. A total of 16 DEGs, including four pistil development-related genes (*Bra026791, Bra020391, Bra029154* and *Bra012128*) and 12 floral organ development-related genes (*Bra001764, Bra031065, Bra022475, Bra000468, Bra010052, Bra007491, Bra000438, Bra001268, Bra019903, Bra009921, Bra003296*, and *Bra021235*) were selected for qRT-PCR analysis (**Supplementary Table S9**). As shown in **Figure 8**, the gene expression patterns obtained by qRT-PCR showed the similar trends as those of RNA-Seq data, thus supporting the reliability of our transcriptome analysis.

## Discussion

In this study, we identified the *fsm* mutant in Chinese cabbage, which exhibited stable inheritance. Based on a comparison with ‘FT,’ we speculate that the mutant gene in *fsm* likely influences ovule development, which further affects normal fertilization process, eventually leading to female sterility. Comparative transcriptome analysis of ‘FT’ and *fsm* showed that a number of DEGs are related to pistil development, and numerous DEGs involved in floral organ development were also identified. Further investigating these DEGs may increase our understanding of the regulatory mechanisms underlying female sterility.

The previous study has reported that the microspore culture enjoyed the characteristic of spontaneous doubling, requiring no artificial doubling treatment, and a high percentage of spontaneous diploids were found in *Brassica campestris* (Zhang and Ting Guan, 1993). The research on the combination of isolated microspore culture and EMS mutagenesis indicated that the frequency of spontaneous diploids were approximately 76.4% in Chinese cabbage (Huang et al., 2016a). In this study, the *fsm* mutant is different from previously reported mutants (Huang et al., 2015, 2016b). In general, the mutation occurred during the haploid microspore period, however, the mutation of the *fsm* mutant occurred during the spontaneous diploid period, and the mutant gene *fsm* was recessive. Given this, the *fsm* mutant exhibited the same visible phenotype as wild-type line ‘FT’ in the M_0_ generation, and segregation of characters appeared in the M_1_ generation, which exhibiting the mutant character.

The female-sterile mutants represent important materials for exploring floral organ-specific gene regulation and function. In the present study, genetic analysis indicated that the mutant phenotype of *fsm* is controlled by a single recessive nuclear gene, however, fine mapping of *fsm* remains to be performed. The results of gene mapping and our transcriptome analysis could be combined to further investigate candidate genes in *fsm*, especially the DEGs identified between ‘FT’ and *fsm*, as well as SNPs specifically detected in *fsm* based on the RNA-Seq results. The DEGs with most dramatically reduced expression levels in the *fsm* mutant could be candidates of the mutant gene, which would be responsible for the pistil developmental phenotype in *fsm*. Further investigating the *fsm* gene may help reveal the regulatory mechanisms underlying pistil development in Chinese cabbage. In addition, the developed SNPs markers represent a rich source of valuable molecular markers, which are widely used for genetic mapping and genetic diversity analysis in plants (Blair et al., 2013; Frascaroli et al., 2013). In this study, finding SNPs between ‘FT’ and *fsm* is especially useful, as some SNPs specifically detected in *fsm* may be directly related to the mutant phenotype.

Morphological observations suggested that the abnormal ovules in *fsm* likely influenced normal fertilization process, ultimately leading to female sterility. Therefore, identifying genes involved in pistil development would facilitate the analysis of female sterility. *PRETTY FEW SEEDS2* (*PFS2*) is primarily expressed in developing primordia, and its transcripts are most abundant in developing ovules. *PFS2* encodes a homeodomain protein that plays a prominent role during ovule patterning by regulating the differentiation of megaspore mother cells and cell proliferation of maternal integuments (Park et al., 2004). *AGAMOUS* (*AG*) can regulate ovule development and floral development (Becker and Theissen, 2003), and the research has showed that the ovule development is closely related to the level of *PFS2* activity, which can repress *AG* expression in *Arabidopsis thaliana* (Park et al., 2005). In the present study, the up-regulation of *PFS2* (*Bra026791*) detected in *fsm* vs. ‘FT’ may inhibit the *AG* expression, and thus affect ovule development of *fsm*. The female gametophyte development is a complicated process, and numerous genes are involved in its regulation in *Arabidopsis thaliana* (Wang et al., 2012). The *temperature-induced lipocalin* (*TIL*), which is mainly expressed in the embryo sacs of ovules, plays an essential role in female gametophyte development. Mutation of *TIL* causes ovule abortion and sometimes seed abortion, ultimately leading to low seed set (Chang et al., 2014). In the MADS-box gene family, *AGAMOUS* (*AG*) gene plays an important role in regulating floral carpel and ovule development (Alvarez-Buylla et al., 2000; Becker and Theissen, 2003). The related studies indicated that the *AGAMOUS-LIKE 6* (*AGL6*) gene played an essential role in the floral development (Rijpkema et al., 2009). In *Arabidopsis thaliana, AGL6* gene was mainly expressed in the ovule and not expressed in the stamens (Schauer et al., 2009), however, the *AGL13* gene was expressed in both ovule and stamens, and had the function of regulating stamen and pistil development (Hsu et al., 2014). In addition, the *AGL23* gene had an effect on the female gametophyte development (Colombo et al., 2008). The gene function of *AGL62* was similar to *AGL61*, which interacted with *AGL80*, and they were jointly associated with the differentiation of central cells in the female gametophyte (Portereiko et al., 2006; Bemer et al., 2008; Kang et al., 2008). The basic helix-loop-helix (bHLH) transcription factors, *HECATE 1* (*HEC1*), *HEC2* and *HEC3* genes are involved in the transmitting tract formation and stigma development, and the *HEC* activity is very necessary in the developing gynoecium in *Arabidopsis thaliana* (Gremski et al., 2007). In addition, the *HEC* genes can regulate both auxin and cytokinine signaling during gynoecium development (Schuster et al., 2015). Therefore, the interaction of these DEGs related to pistil development may influence ovule development, ultimately resulting in the pistil abortion phenotype of *fsm*.

In this study, the *fsm* plants not only exhibited pistil abortion, but the floral organs were also relatively smaller compared to the wild-type line ‘FT.’ The floral organ size is closely related to the floral morphology and function in plants (Horiguchi et al., 2006). The two cellular processes, cell proliferation and cell growth, can control the final size of floral organs during the process of floral development (Horiguchi et al., 2006; Krizek and Anderson, 2013). The research has shown that the *ARGOS* gene can control the floral organ size in *Arabidopsis thaliana*, with the up-regulation or down-regulation of *ARGOS* gene, the size of floral organs would be increased or decreased accordingly (Hu et al., 2003). The homologous gene of *ARGOS* has been isolated from Chinese cabbage, and the overexpression of *ARGOS* gene can make a significant increase in the leaves and floral organs in *Arabidopsis thaliana* (Hu et al., 2003, 2006; Feng et al., 2011). Based on the results presented here, the *ARGOS* gene was up-regulated in *fsm* vs. ‘FT’ and specifically expressed in *fsm*, however, the floral organs were relatively smaller in *fsm*. The result was opposite to the previous studies (Hu et al., 2003, 2006). In this study, except for the *ARGOS* gene, other DEGs involved in the floral development were also found, such as F-box family protein, *FT* and *MYB* family transcription factors. Therefore, we speculated that the interaction and regulation of these DEGs may result in the differences between ‘FT’ and *fsm* in floral organ sizes.

Among the genes differed in expression between ‘FT’ and *fsm*, approximately one third of genes which were annotated as unknown function. These genes might be the potential candidates to be involved in the pistil development, especially for the SEGs. The genes expressed exclusively in *fsm* may lead to the female sterility by inhibiting the pistil development. On the other hand, the genes only expressed in ‘FT’ were detected, which may have a role in the pistil development. Therefore, the actual functions of these genes remain to be further studied.

The previous studies indicated that heat or cold stress response-related proteins were associated with the pistil development in plants (Zinn et al., 2010). In *Arabidopsis thaliana*, heat-stress treatment can increase the number of aborted ovules and reduce the ovule numbers (Whittle et al., 2009). The related studies showed cold stress can reduce ovule fertilization and ovule viability, and thus affect the pistil function (Srinivasan et al., 1999; Thomashow, 1999). In our study, a number of DEGs related with heat or cold temperature stress were identified, such as heat shock proteins (HSP; *Bra018216, Bra006697, Bra002539*, and *Bra020295*) and late embryogenesis abundant (LEA) proteins (*Bra022950, Bra027219*, and *Bra030494*). Besides, the GO term enrichment analysis showed the GO term “response to heat” (GO: 0009408; 16 DEGs, 1.5%) was detected. Therefore, we speculated that these genes associated with hot or cold temperature stress may play important roles in the pistil abortion in *fsm*.

The KEGG pathway analysis revealed that a total of eight KEGG pathways were significantly enriched, of which, 103 (10.85%) DEGs were involved in the Plant hormone signal transduction pathway (ko04075). In plants, flower development was strongly influenced by hormonal regulation (Rudich et al., 1972). The previous studies indicated that the genes involved in the hormone signaling may play important roles in plant sex determination (Chandler, 2011). In recent years, several different transcriptome analyses showed that numerous hormone-related genes were differently expressed between different flower types, which further indicated hormones had roles in the sexual differentiation and development of floral organ (Wu et al., 2010; Ramos et al., 2014; Rocheta et al., 2014; Sobral et al., 2016). For example, auxin enjoyed a critically regulatory function in the process of floral growth and development in plants (Aloni et al., 2006; Cheng and Zhao, 2007). The recent studies indicated that cytokinine facilitates cell proliferation in early reproductive tract development and regulates reproductive meristems and ovule formation (Bartrina et al., 2011; Marsch-Martínez et al., 2012a). The interaction between auxin and cytokinine has been demonstrated to have a role in the gynoecium morphogenesis (Marsch-Martínez et al., 2012b). Another hormone, ethylene, was strongly relevant to the sex determination (Byers et al., 1972). The ethylene was thought to be essential for the process of sex determination in several species, such as cucumber and melon (Guo et al., 2010; Gao et al., 2015). In this study, the genes involved in the auxin signaling, cytokinine signaling and ethylene signaling were differentially expressed and had relatively high expression levels. Further study of these genes related to the hormone signal transduction may contribute to elucidate the female organ determination in Chinese cabbage.

## Conclusion

We performed a systematic morphological investigation of *fsm*, followed by comparative transcriptome analysis between ‘FT’ and *fsm*. The results provide a comprehensive view of the expression profiles of genes involved in pistil development, which may help uncover the molecular mechanisms determining the phenotypic differences between these lines. Further studies of the functions of DEGs involved in pistil development should increase our understanding of female sterility. Our results provide a solid foundation for the further functional characterization of genes associated with the pistil development in Chinese cabbage.

## Database Linking

The transcriptome sequencing data were deposited in the NCBI Gene Expression Omnibus (GEO) Database under accession number GSE76917.

## Author Contributions

HF and ZL conceived and designed the research. SH, CL, DL, and RY performed the research. SH, LH, XL, and WL analyzed the data. SH wrote the manuscript. HF revised the manuscript. All authors discussed the results and approved the final manuscript.

## Conflict of Interest Statement

The authors declare that the research was conducted in the absence of any commercial or financial relationships that could be construed as a potential conflict of interest.

## Footnotes

1 http://brassicadb.org/brad/

2 http://www.geneontology.org/

3 http://www.genome.ad.jp/kegg/

## Supplementary Material

TABLE S1 **List of all genes detected in the ‘FT’ and *fsm* libraries (XLS)**.

TABLE S2 **Summary of SNPs detected in the ‘FT’ library (XLSX)**.

TABLE S3 **Summary of SNPs detected in the *fsm* library (XLSX)**.

TABLE S4 **The SNPs specifically detected in the ‘FT’ library (XLS)**.

TABLE S5 **The SNPs specifically detected in the *fsm* library (XLS)**.

TABLE S6 **List of DEGs identified in the *fsm* vs. ‘FT’ comparison (XLS). Gene IDs and expression levels of DEGs are shown**.

TABLE S7 **List of SEGs identified in the *fsm* vs. ‘FT’ comparison (XLS)**.

TABLE S8 **Significantly enriched GO terms identified in the *fsm* vs. ‘FT’ comparison (XLS)**.

TABLE S9 **Primer sequences used for qRT-PCR expression analysis (XLS)**.
